# *In vitro* selective cytotoxicity of the dietary chalcone cardamonin (CD) on melanoma compared to healthy cells is mediated by apoptosis

**DOI:** 10.1371/journal.pone.0222267

**Published:** 2019-09-25

**Authors:** Lena Berning, Lisa Scharf, Elif Aplak, David Stucki, Claudia von Montfort, Andreas S. Reichert, Wilhelm Stahl, Peter Brenneisen

**Affiliations:** Institute of Biochemistry and Molecular Biology I, Medical Faculty, Heinrich-Heine University, Düsseldorf, Germany; Chung Shan Medical University, TAIWAN

## Abstract

Malignant melanoma is an aggressive type of cancer and the deadliest form of skin cancer. Even though enormous efforts have been undertaken, in particular the treatment options against the metastasizing form are challenging and the prognosis is generally poor. A novel therapeutical approach is the application of secondary plant constituents occurring in food and food products. Herein, the effect of the dietary chalcone cardamonin, inter alia found in *Alpinia* species, was tested using human malignant melanoma cells. These data were compared to cardamonin treated normal melanocytes and dermal fibroblasts representing healthy cells. To investigate the impact of cardamonin on tumor and normal cells, it was added to monolayer cell cultures and cytotoxicity, proliferation, tumor invasion, and apoptosis were studied with appropriate cell biological and biochemical methods. Cardamonin treatment resulted in an apoptosis-mediated increase in cytotoxicity towards tumor cells, a decrease in their proliferation rate, and a lowered invasive capacity, whereas the viability of melanocytes and fibroblasts was hardly affected at such concentrations. A selective cytotoxic effect of cardamonin on melanoma cells compared to normal (healthy) cells was shown *in vitro*. This study along with others highlights that dietary chalcones may be a valuable tool in anticancer therapies which has to be proven in the future *in vivo*.

## Introduction

The worldwide incidence of all forms of skin cancer has significantly risen over the last decades, and especially the malignant melanoma is responsible for the majority of skin cancer deaths [1–3]. The incidence of cutaneous melanoma varies among populations. The disease occurs very frequently with fair-skinned Caucasian populations in Australia and New Zealand followed by North America and Northern/Western Europe [4]. The cause of melanoma is not precisely known, but there is no doubt so far, that the major risk factor is over exposure to ultraviolet radiation (UV) due to intensive sun bathing, increased outdoor recreational activities, and clothing that exposes more skin [5–7]. Primarily, the classical treatment of melanoma comprises an adequate surgical excision followed by radiotherapy- and/or chemotherapy-based regimes, if the tumor already metastasized. Recently, targeted therapies using inhibitors directed against signaling components and immunotherapies with monoclonal antibodies e.g. against immune checkpoint receptors are gaining ground [8–11]. In addition, the medical use of nanoparticles either as carrier of drugs to specific cellular targets or as so-called nanopharmaceuticals is another possibility for the treatment [12–14]. However, many of these therapies result in drug resistance and may cause severe side effects because normal (healthy) cells are affected [15–17]. The overall rates of some types of cancer including melanoma did not change substantially or is rather increasing since former U.S. President Nixon declared the “War on Cancer” more than 45 years ago [18]. Studies on secondary plant constituents awake the hope to identify new (active) chemical compounds with protective or antiproliferative properties [19,20]. Chemoprevention by dietary compounds with antioxidant capacities or effects on proliferation/growth-promoting signaling pathways is of considerable interest. In that context, dietary chalcones with the core structure 1,3-diphenyl-2-propenone representing a group of the polyphenolic family have been described to exhibit an interesting spectrum of biological/biochemical activities comprising antibacterial, anti-inflammatory, antioxidative, and immunomodulatory potential as well as both chemopreventive and chemotherapeutic activities [21]. Edwards et al. [22] identified chalcones as a new class of antimitotic agents. They showed that microtubule assembly and the completion of cell division of HeLa tumor cells was prevented by one of the studied synthetic chalcones. Numerous other synthetic chalcones have been tested and showed antitumorigenic, apoptosis-stimulating properties on melanoma and other cancer types [23–26]. One of the best described naturally occurring chalcones with widespread biological activities is xanthohumol (XN) [27], a chalcone occurring in beer hops. An apoptosis-initiating and angiogenesis-suppressing effect of XN was reported for colon cancer cells, breast cancer cells, and pancreatic adenocarcinoma cells to name but a few [21,28–30]. Moreover, a xanthohumol mediated increase in oxidative stress triggered benign prostatic hyperplasia cells into apoptosis [31]. As can be seen by the increasing number of publications, the alpha,beta-unsaturated chalcone cardamonin (CD) [32], found in cardamom spice and in Alpinia species, is now subject of interest in medicine [21,33]. In addition to its potential antioxidant, anti-inflammatory, and anti-infectious activity summarized, for example, by Gonçalves et al. [33], CD has been described to have antineoplastic activity. CD inhibits proliferation and metastasis of the non-small-cell lung cancer cell lines A549 and H460 [34] and induces autophagic cell death in SKOV3 ovarian carcinoma cells [35]. Furthermore, migration and invasive capacity is downregulated in CD-treated triple negative breast cancer cell line BT-549 [36] and the androgen-independent DU145 prostate cancer cell line [37]. In addition, CD induces apoptosis in CD133+ glioblastoma stem cells [38] and HCT116 colon cancer cells [39]. Even though CD appears to have antitumor potential on numerous cancer types, nothing is known about cytotoxic and anti-invasive effect of CD on metastatic melanoma. Additionally, no information is available on the selectivity of chalcones regarding the inhibition of tumor growth in comparison to their effects on normal (healthy) cells. In the present in vitro study, the effect of CD on the human malignant melanoma cell line A375 was tested and compared with the effect on normal human epidermal melanocytes (NHEM), as model of the healthy counterpart, and on normal human dermal fibroblasts (NHDF) used as model for stromal cells as these cells are often found at the invasion front of skin tumors [40]. To our knowledge, this is the first study showing that the alpha,beta-unsaturated chalcone CD has a selective cytotoxic, antiproliferative, anti-invasive, and proapoptotic impact on melanoma cells *in vitro*, whereas normal (healthy) cells are not affected by the same concentrations indicating the potential anticancer activity of CD.

## Materials and methods

### Reagents

**Fig 1:**
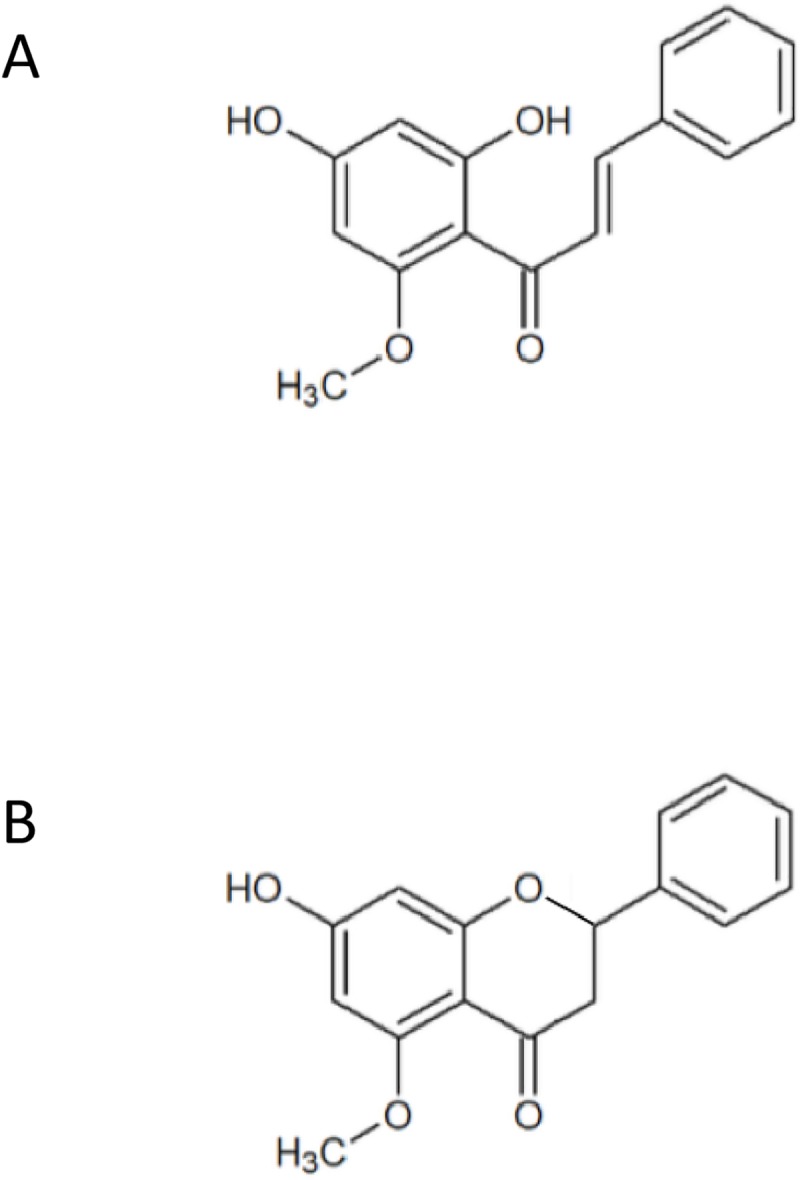
Chemical structure of the chalcone cardamonin (CD) (A) and the flavanone alpinetin (B).

All chemicals were obtained from Sigma-Aldrich (Darmstadt, Germany) including Dulbecco modified Eagle’s medium (DMEM), unless otherwise stated. 4-hydroxynonenal was purchased from Biomol (Hamburg, Germany), cardamonin and alpinetin (Fig 1) from Phytolab (Verstenbergsgreuth, Germany). Penicillin/Streptomycin was from Biochrom and Glutamax from Gibco (Darmstadt, Germany). The fetal bovine serum (FBS) was from Pan-Biotech (Aidenbach, Germany). The melanocyte growth medium and the growth medium supplement mix were purchased from PromoCell (Heidelberg, Germany). Hank’s Balanced Salt Solution (HBSS) was from Gibco (Darmstadt, Germany). DMSO was obtained from Roth (Karlsruhe, Germany). Recombinant human TGFß1 was purchased from R&D Systems (Wiesbaden, Germany) and the pan caspase inhibitor z-VAD-FMK from Santa Cruz Biotechnology (Heidelberg, Germany).

### Cell culture

Human melanoma cell line A375 (CRL-1619) was purchased from the American Type Culture Collection (ATCC, Virginia, USA). Normal human dermal fibroblast (NHDF; C-12300) and normal human epidermal melanocytes (NHEM; C-12400) were from PromoCell (Heidelberg, Germany). Under routine conditions, A375 and NHDF were cultured in Dulbecco’s Modified Eagle’s (DMEM, low glucose (5.5 mM)), supplemented with 10% fetal bovine serum (FBS), streptomycin (100 μg/ml), penicillin (100 U/ml) and GlutaMAX^TM^ (L-alanyl-L-glutamine source, 2 mM). Under starvation (serum-free) conditions, the cells were cultured in DMEM including high glucose (25 mM) and 2 mM GlutaMAX^TM^. NHEM were cultured in melanocyte growth medium, supplemented with growth medium supplement mix, streptomycin (100 μg/ml) and penicillin (100 U/ml). All cells were cultured at 37°C and 5% CO_2_ in a humidified atmosphere. For the experiments, subconfluent cells (70–80% confluence) were used. The substances were directly added to the cells.

### Cell viability assays

#### MTT assay

Cell viability was measured by the MTT (3-(4,5-dimethylthiazol-2-yl)-2,5-diphenyltetrazolium bromide) assay [41]. Briefly, the activity of mitochondrial dehydrogenase results in formation of a purple formazan dye which can be detected by absorbance. The assay was performed using 24 well plates. Subconfluent cells were treated with different concentrations of DMSO dissolved CD and 4-hydroxynonenal, respectively, or 2 mM H_2_O_2_ (positive control). Subsequently, the cells were washed with PBS once, MTT solution (0.5 mg/ml) was added to the cells and the cells were incubated between 0.5 and 2 h. The MTT was removed and the cells were washed 1x with phosphate-buffered saline (PBS). The formazan was extracted with 0.5 ml DMSO per well. Absorbance was measured with a plate reader at 570 nm. The untreated control was set to 100%. IC_50_ values were calculated with Prism 6 (GraphPad Software Inc., California, USA).

#### Sulforhodamine B (SRB) assay

The assay based on the pH dependent staining of total proteins using SRB as dye [42]. The assay was performed using 24 well plates. After incubation with different concentrations of the substances, the medium was removed and the cells were washed 1x with PBS, fixed with a TCA solution (10% w/v) for 60 min at 4°C, washed 5x with dH_2_O, and dried overnight at RT. After staining with 300 μl SRB solution (0.4% w/v in 1% acetic acid) for 15 min at RT, the cells were 5x washed with 1% acetic acid and dried at RT. For extraction of the dye, 400 μl TRIS-Base (10 mmol/l) was added and the plate was gently rotated for 5 min. Finally, the absorbance was measured at 490 nm with a microplate reader.

### Proliferation assay

DNA synthesis was determined by measuring bromodeoxyuridine (BrdU) incorporation into DNA with the BrdU proliferation assay following the manufacturer’s instructions (Cell Signaling Technology, Massachusetts, USA).

### Preparation of conditioned medium (CM)

The conditioned medium was obtained from human dermal fibroblasts (CM^NHDF^) and myofibroblasts (CM^MF^) as previously described [13]. Briefly, seeded NHDF were grown to subconfluence (about 70% confluence) in 175-cm^2^ culture flasks in serum-containing medium. After washing in phosphate-buffered saline (PBS), the cells were treated with 5 ng recombinant transforming growth factor-beta 1 (rTGFß1) per ml (for generation of myofibroblasts) or without rTGFß1 in serum- free DMEM for 48 h. This medium was removed, and after washing in PBS, all cells were incubated in 15 ml serum-free DMEM for further 48 h before collection of the CM^NHDF^ and CM^MF^.

### Invasion assay

After 48 h incubation with CD, A375 melanoma cells were harvested and seeded at a density of 4 x 10^4^ in serum-free DMEM in the upper transwell culture chambers (8 μm pore size) coated with Matrigel (BD Biosciences). Conditioned medium of fibroblasts (CM^NHDF^) or myofibroblasts (CM^MF^) was used as a chemoattractant. After 48 h incubation, the invaded cells on the lower membrane surface were fixed with methanol and stained with Coomassie Blue solution (0.05% Coomassie Blue; 20% MeOH; 7.5% acetic acid). The invaded cells were counted and each sample was analyzed in triplicate and a total of three independent experiments was performed (n = 3).

### HPLC—Cellular uptake of CD and detection of alpinetin

Cellular uptake of CD and the CD cyclization product alpinetin were determined by HPLC after treatment of cells with CD. The cells were grown to subconfluence in Ø 6 cm-culture dishes. After incubation with 10 μM CD at different times, 0.5 ml of the supernatant was stored in liquid nitrogen. Cells were washed with PBS twice and harvested in 0.5 ml PBS. Samples were centrifuged at 1700xg for 6 min at 4°C. Cell pellets were resuspended in 0.1 ml ice cold methanol and centrifuged at 14000xg for 10 min at 4°C. For HPLC analyses, 50 μl of the methanol extracts were injected, and the CD concentration was quantified using a standard curve. The HPLC system consisted of a Merck-Hitachi L-7100 pump connected with a Merck-Hitachi diode array detector (DAD) (Merck-Hitachi L-7450) and a data registration system. Wavelength range was 220–400 nm, CD was analyzed at 342 nm, and alpinetin at 284 nm. A Waters CORTECS UPLC column, C-18 (4.6 mm x 150 mm, 2.7 μm) from Waters (Massachusetts, USA) was used as stationary phase. HPLC was performed with a mobile phase of 60% water, 37% acetonitrile and 3% HAc (v/v/v) at a flow rate of 1.0 ml/min (0–5 min) and a mobile phase of 47% water, 50% acetonitrile and 3% HAc (v/v/v) at a flow rate of 1.0 ml/min (5–20 min). Retention time of CD was around 9 min and for alpinetin around 4 min. The cell pellet was lysed in 50 μl SDS lysis buffer and the protein concentration was determined by using the DC^TM^ Protein Assay Kit (Bio-Rad, California, USA). Finally, the concentration of CD was set in relation to the protein amount.

### Measurement of intracellular ROS

The level of intracellular reactive oxygen species (ROS) was determined using 2’,7’-dichlorodihydrofluorescein diacetate (H_2_DCF-DA), which intracellularly is oxidized by ROS to the fluorescent DCF. The assay was performed using 24 well plates. Cells were grown to subconfluence and incubated with 100 μM H_2_DCF-DA in HBSS for 30 min. Subsequently, the solution was removed, cells were washed twice with HBSS and 2 or 5 μM CD was added in 0.5 ml HBSS. DCF fluorescence was measured at an excitation wavelength of 485 nm and emission wavelength of 520 nm in 5 min intervals with a plate reader.

### SDS PAGE and western blotting

For SDS PAGE and Western blotting [43], cells were lysed after incubation with CD in 1% SDS with 1:1000 protease inhibitor cocktail (Sigma, Taufkirchen, Germany). After sonication, the protein concentration was calculated using the DC^TM^ Protein Assay Kit. Sample buffer (40% glycerol, 20% ß-mercaptoethanol, 12% SDS, 0.4% bromphenol blue) was added to equal amounts of protein (20 μg), and after heating, the samples were subjected to 12% (w/v) SDS-polyacrylamide gels. After electroblotting of proteins onto polyvinylidene difluoride (PVDF) membranes (GE Healthcare, Solingen, Germany), the blot was developed using the ECL-system (Cell Signaling Technology) and quantified by the image processing program Image J (Wayne Rasband, NIH) Primary antibodies (1:1000 dilution) were used: rabbit monoclonal anti-human/mouse HO-1 from Abcam (Cambridge, UK); rabbit monoclonal anti-human cleaved/active caspase 3 (Asp175) and rabbit monoclonal anti-human cleaved PARP (Asp214) from Cell Signaling Technology (Massachusetts, USA), anti-human α-tubulin from Sigma-Aldrich; secondary antibodies (1:20000 dilution) were: horseradish peroxidase (HRP)-conjugated goat anti-rabbit IgG from Dianova (Hamburg, Germany) and HRP-counjugated rabbit anti-mouse IgG from Dako (Glostrup, Denmark). α-tubulin was used as an internal loading control.

### Statistical analysis

Means were calculated from at least three independent experiments, unless otherwise stated. Data are presented as mean ± standard error of the mean (SEM). Statistical analysis was performed by Student’s t-test or ANOVA with Dunnett’s post-hoc test with *p<0.05, **p<0.01 and ***p<0.001 as levels of significance.

## Results

### CD selectively lowers viability of human melanoma cells compared to human melanocytes and dermal fibroblasts

**Fig 2:**
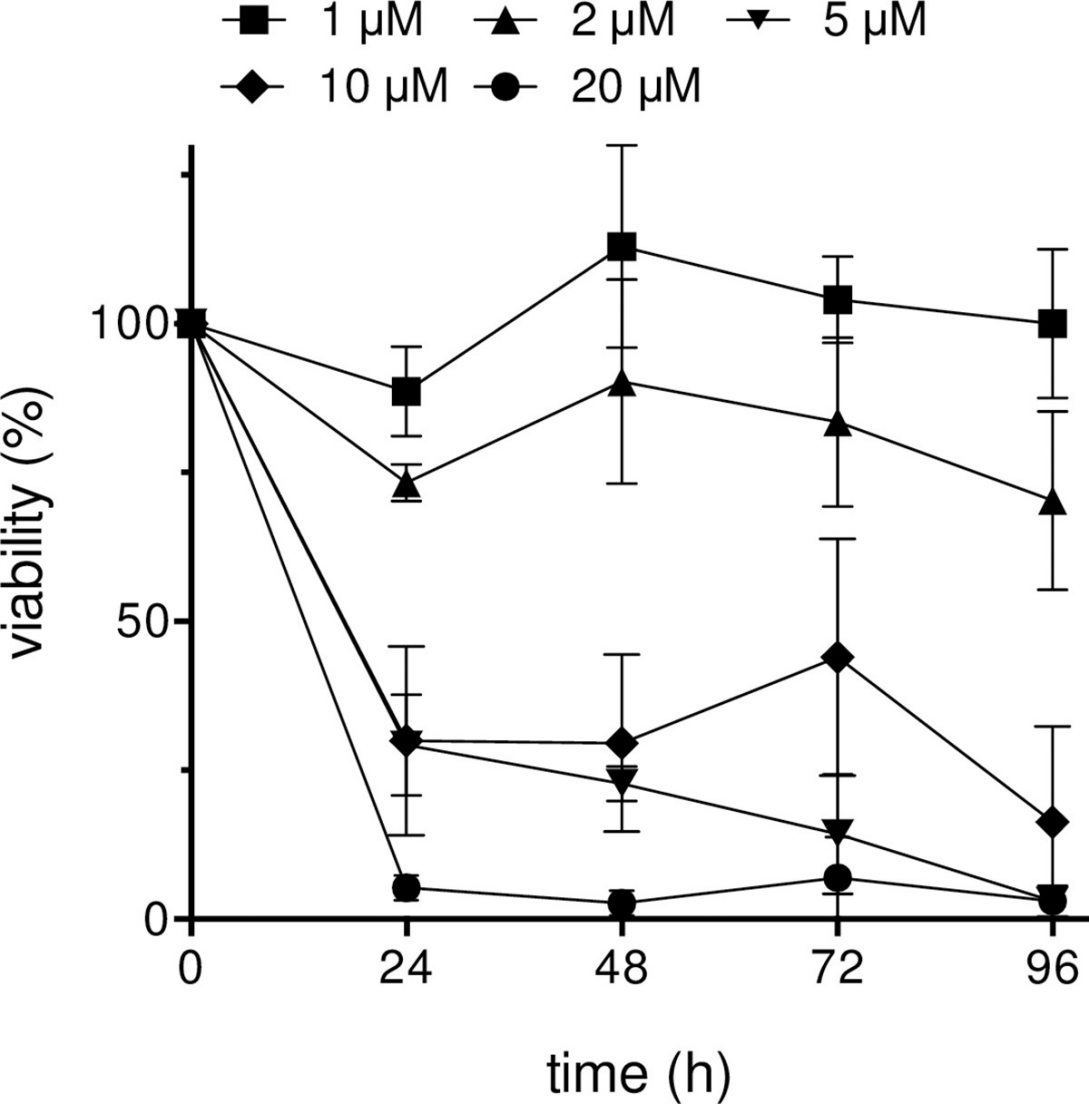
Significant decrease of the viability of A375 melanoma cells after CD treatment. Subconfluent melanoma cells were incubated with different CD concentrations for 24, 48, 72, and 96 h and viability measured with the MTT assay. The viability is shown as a percentage of mock-treated control (ct), which was set at 100%. Three (n = 3 for 0, 24, 48, 96 h) and four (n = 4 for 72 h) independent experiments were performed. For each experiment, 3 different dishes were measured and mean and standard deviations were calculated according to the Gaussian error propagation law.

At concentrations of 5, 10 and 20 μM CD (Fig 1) the viability of the A375 melanoma cells in subconfluent monolayer cultures was significantly lowered below 50% already after 24 h treatment compared with mock-treated cells (control, ct) which were set at 100% (Fig 2). The strongest cytotoxic effect was observed with 20 μM CD as only a fraction of about 5% tumor cells still survived 24 h after treatment, whereas at concentrations of 5 and 10 μM CD a higher percentage of tumor cells was still alive between 24 and 72 h. After a 96 h treatment, these concentrations showed similar results. In contrast, the viability of the melanoma cells was about 70% and 100% after 96 h treatment at low doses of 2 and 1 μM CD, respectively (Fig 2, S1 Fig). Although it seems that a concentration of 5 μM CD led to a higher toxicity between 48 to 96 h compared to 10 μM CD, the evaluation of the standard deviation showed no significance (p>0.05). From these data, we conclude that concentrations greater than 5 μM CD result in a significant damage of the melanoma cells at a certain time after treatment at which a dose-dependent toxicity is rather difficult to evaluate.

**Fig 3:**
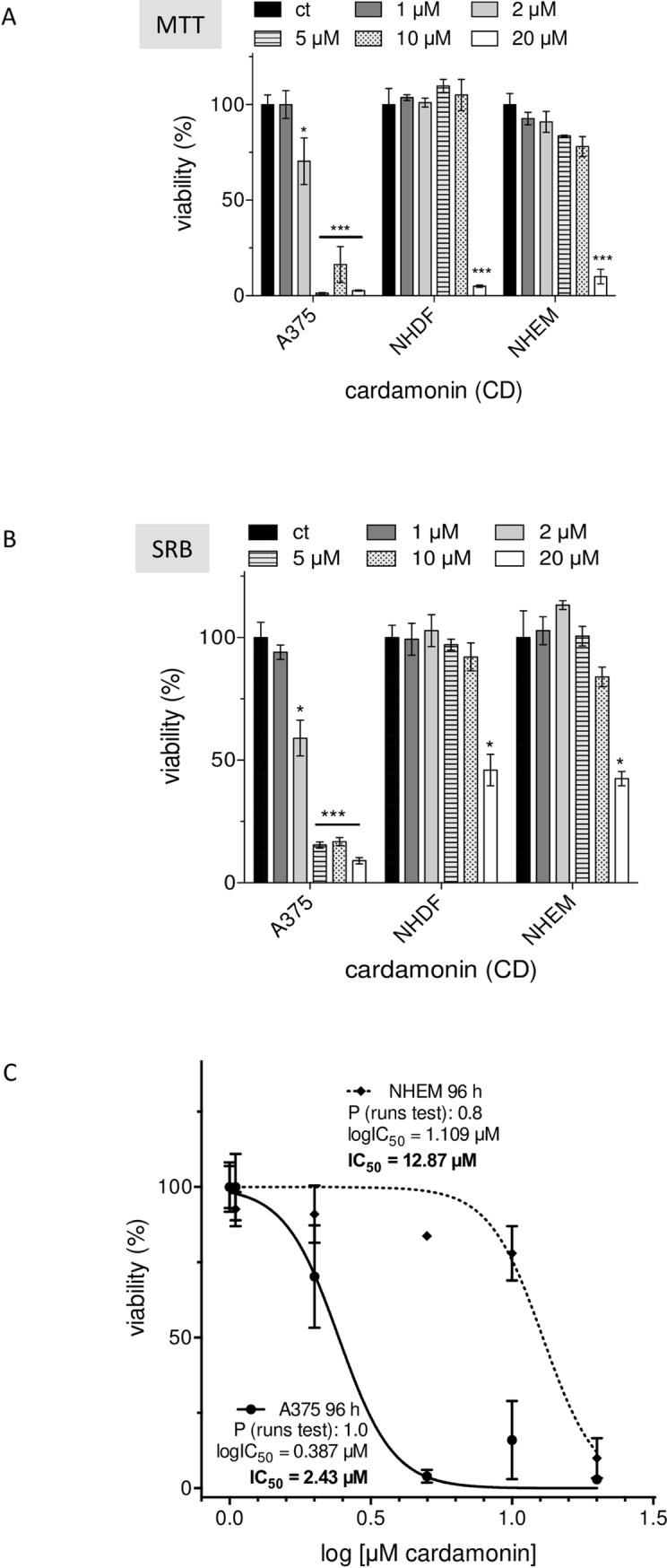
Selective cytotoxicity of CD on A375 melanoma cells. Melanoma cells, normal human dermal fibroblasts (NHDF), and normal epidermal melanocytes (NHEM) were incubated with different concentrations of CD for 96 h and viability was measured with the MTT (A) or SRB assay (B). The percentage of cell viability of the mock-treated control (ct), which was set at 100%, is presented (n = 3). In addition, IC_50_ values of melanoma cells versus melanocytes were calculated by non-linear curve fit analysis using Prism software (**Graph**Pad, San Diego, USA) with R^2^ >0.9 and P >0.5 (runs test) as parameters of goodness-of-fit (C). The level of significance versus ct was calculated with Student’s t-test or ANOVA (Dunnett’s test) with ***p<0.001, and *p<0.05.

Furthermore, the effect of CD on the viability of normal human epidermal melanocytes (NHEM) and normal human dermal fibroblasts (NHDF) was measured with the MTT assay at 96 h after treatment the three cell types with different concentrations of CD. The MTT assay is based on measurement of the metabolic activity of cells. This assay quantifies viable cells through their ability to reduce 3-(4,5-dimethylthiazol-2yl)-2,5-diphenyltetrazolium bromide, a soluble yellow tetrazolium salt to a blue-purple formazan precipitate which requires the activity of the mitochondrial succinate dehydrogenase [44,45]. As the MTT formazan *per se* is cytotoxic over time resulting in false-positive (false-negative) results, a second viability assay was used to verify the MTT data. The sulphorhodamine B (SRB) assay was applied providing a good linearity with cell number and being independent of intermediary metabolism. SRB is an anionic dye, which binds to basic amino acid residues in fixed cells to provide a sensitive index of cellular proteins [46]. Both assays showed in tendency same results (Fig 3A, MTT; Fig 3B, SRB). Incubation the cells with 2, 5, and 10 μM CD significantly decreased the viability of the melanoma cells, whereas these concentrations showed no cytotoxic effect on NHEM and NHDF compared to mock-treated cells (ct). The lowest concentration of CD had no effect on all three cell types. In contrast, the application of the highest concentration of 20 μM CD resulted in a significant lowered cell viability for all three cell types (Fig 3A and 3B). These data are reflected by the IC_50_ values of the melanoma cells and NHEM as normal counterpart (Fig 3C) based on MTT data after 96 h treatment. The IC_50_ values were calculated by nonlinear curve fit analysis and evaluation of goodness-of-fit (all runs tests >0.5, all R^2^ >0.9) [47]. The IC_50_ for CD-treated A375 melanoma cells was calculated to be 2.43 μM and 12.87 μM CD for NHEM. An IC_50_ of 2.58 μM for the melanoma cells and 18.65 μM CD for NHEM was calculated using the 96 h data of the SRB assay (data not shown). In conclusion, melanoma cells show a significant greater susceptibility against CD compared to normal (healthy) cells turning CD into a promising tumor cell-killing drug, hereinafter referred to as “anticancer drug”.

**Fig 4:**
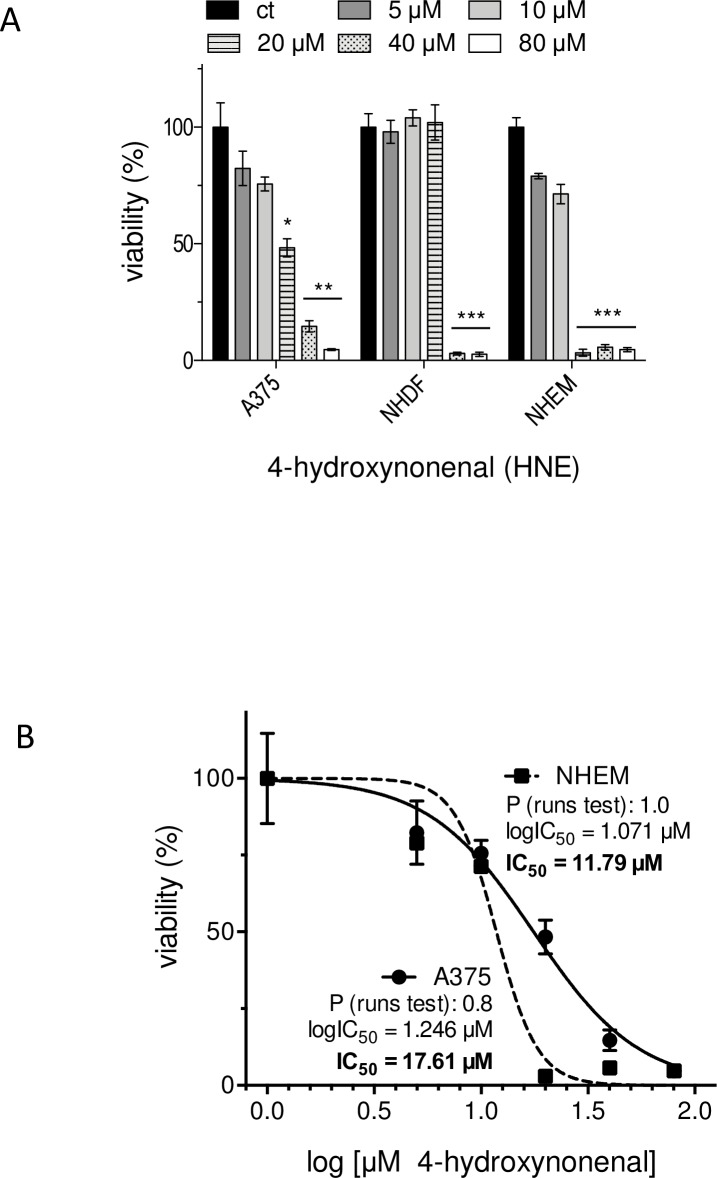
Cytotoxicity of 4-hydroxynonenal (HNE) on A375 melanoma cells and normal (healthy) cells. Melanoma cells, normal human dermal fibroblasts (NHDF), and normal epidermal melanocytes (NHEM) were incubated with different concentrations of HNE for 96 h and viability was measured with the MTT assay (A). The percentage of cell viability of the mock-treated control (ct), which was set at 100%, is presented (n = 3). IC_50_ values of melanoma cells versus melanocytes were calculated by non-linear curve fit analysis using Prism software (**Graph**Pad, San Diego, USA) with R^2^ >0.9 and P >0.5 (runs test) as parameters of goodness-of-fit (B). The level of significance versus ct was calculated with Student’s t-test or ANOVA (Dunnett’s test) with ***p<0.001, **p<0.01, and *p<0.05.

As an alpha,beta-unsaturated carbonyl compound, CD undergoes Michael-Addition reactions with, for example, specific thiol groups of proteins [42]. That could be responsible for the selective cytotoxic effect of CD on the tumor cells as the protein pattern in tumor cells differs from that of normal (healthy) cells [48]. Therefore, we used the alpha,beta-unsaturated 4-hydroxynonenal (HNE) [49] as reference substance and checked the cytotoxicity of HNE on the three cell lines (Fig 4A). Although a dose-dependent increase in cytotoxic activity of HNE was seen in melanoma cells up to 96 h after treatment, a higher survival rate of these tumor cells compared to NHEM was measured at concentrations of 20 and 40 μM HNE indicating that the selective cytotoxic effect of CD on A375 cells does not exclusively depend on the alpha,beta-unsaturated carbonyl structure. In that context, the IC_50_ value (Fig 4B) for HNE is 11.79 μM for NHEM and with 17.61 μM even higher for the A375 tumor cells. For subsequent experiments, 2 μM and 5 μM CD were used which are subtoxic and toxic for melanoma cells, respectively, and non-toxic for normal (healthy) cells.

### CD inhibits proliferation of A375 melanoma cells

**Fig 5:**
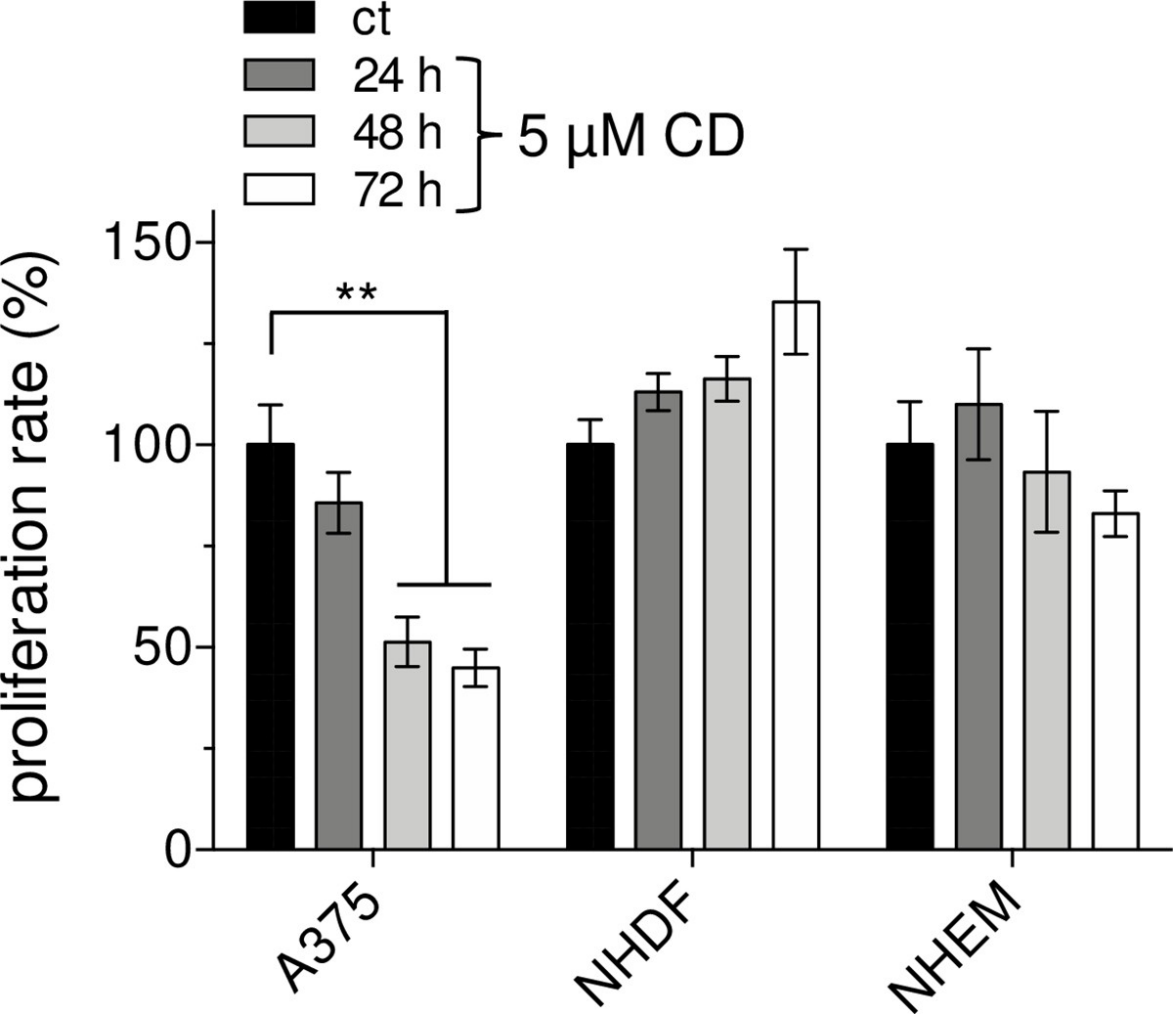
CD selectively lowers proliferation of A375 melanoma cells. Melanoma cells, normal human dermal fibroblasts (NHDF), and normal epidermal melanocytes (NHEM) were incubated with 5μM CD and BrdU incorporation as measure of proliferation was determined at different time points. The proliferation rate of mock-treated cells (ct) was set at 100%. Experiments were performed in triplicate (n = 3). The level of significance was calculated (ANOVA, Dunnett’s test) with **p<0.01.

In order to verify the data on CD cytotoxicity, the proliferation rate of the three cell lines was measured with the thymidine analogue BrdU (Fig 5), widely used as proliferation (DNA replication) and DNA repair marker [50,51]. A concentration of 5 μM CD was used which is non-toxic for NHEM and NHDF, but toxic for the melanoma cells (see Fig 3A and 3B). The cells were incubated in growth factor-free (serum-free), but high glucose (25 mM) and 2 mM L-alanyl-L-glutamine (GlutaMAX^TM^) containing DMEM for various time periods (24, 48, 72 h) and, thereafter, the incorporation of BrdU as measure for proliferation evaluated. As earlier published data show that tumor cells and non-cancerous cells proliferate growth-factor independent with high glucose and a stable, viability increasing glutamine source [52–55], those conditions have been used herein for all cell types to avoid artificial data obtained by exogenous growth factors originating from the serum. Furthermore, it was shown that starvation of tumor cells often results in autophagy-activated cell proliferation [56]. As might be expected, the proliferation rate of the melanoma cells was significantly lowered after 48 and 72 h upon CD treatment compared to the mock-treated cells (ct). However, the decrease in tumor cell proliferation to about 50% by CD is higher than the measured viability data (see Fig 2), that could be explained by DNA repair-mediated BrdU incorporation in addition to DNA replication, as mentioned above. Interestingly, CD did not significantly downregulate the proliferation of NHDF and NHEM under the same cell culture conditions as for the melanoma cells (Fig 5). In conclusion, these data along with the cytotoxicity data confirm that the dietary chalcone CD act selectively on the tumor cells.

### CD lowers the invasive capacity of melanoma cells

**Fig 6:**
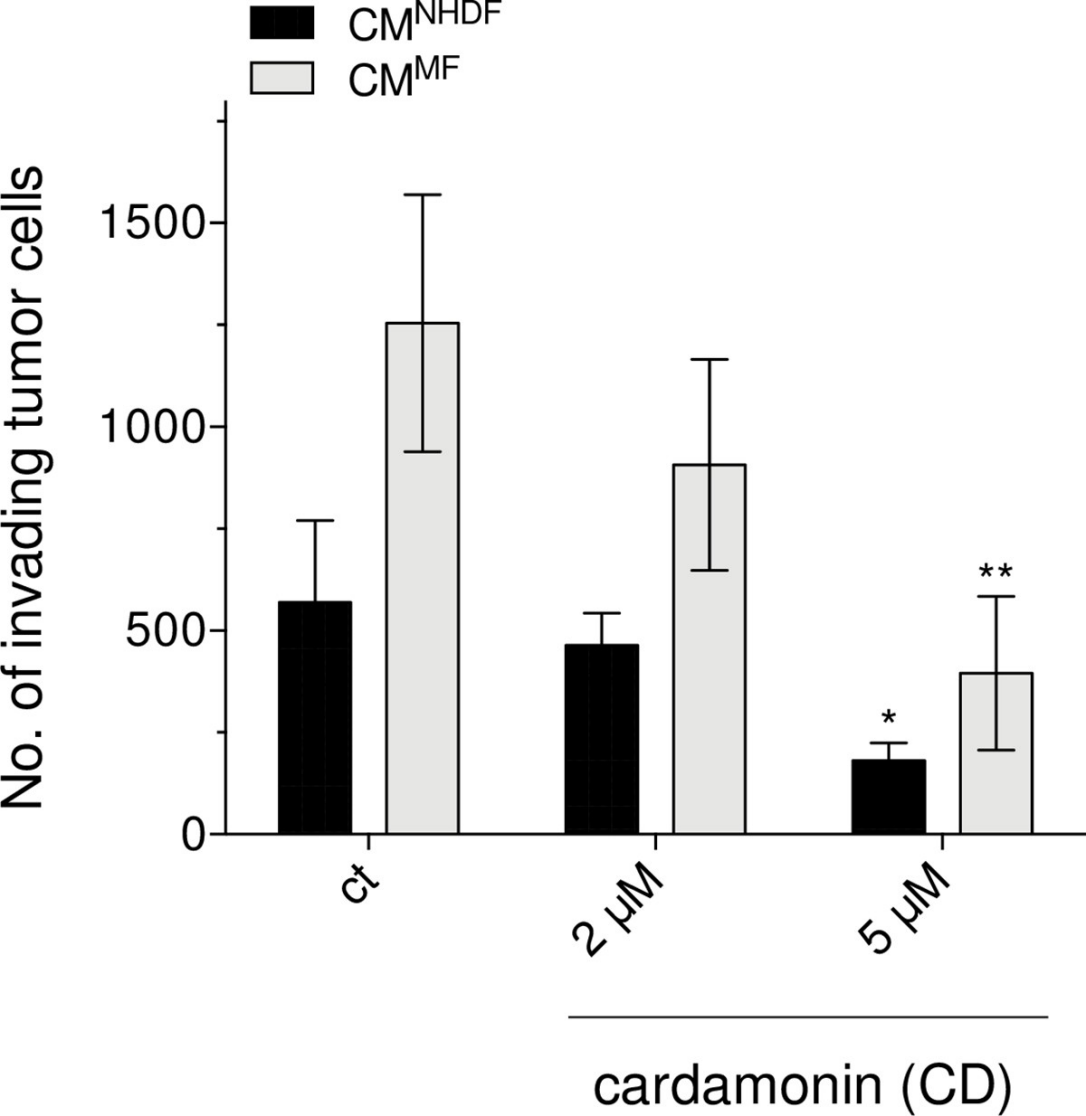
CD lowers invasive capacity of A375 melanoma cells. Subconfluent tumor cells were treated with 2 or 5 μM CD or mock-treated (ct) for 48 h before used for invasion assays. The invasive capacity (matrigel based assay) of the cells was tested with conditioned medium of NHDF (CM^NHDF^) and myofibroblasts (CM^MF^), which were used as chemoattractant (n = 3). **p<0.01 and *p<0.05 versus ct (Student’s t-test).

A major challenge with regard to the treatment of melanoma is the prevention of metastasis. Even though CD was shown to have a dose-dependent cytotoxic effect on the tumor cells, a survival rate of about 10–50% for CD treated melanoma cells was detected at concentrations between 5–10 μM and up to 72 h (see Fig 2). In addition, the proliferation of the CD treated tumor cells was not downregulated to zero (see Fig 5). Therefore, the question was addressed of whether CD can decrease the invasive capacity of that survival fraction. Melanoma cells were incubated with 2 and 5 μM CD for 48 h and the surviving cells were subjected to the Matrigel assay (Fig 6) as described above. The invasive capacity of CD treated cells was compared to mock-treated cells (ct). The conditioned medium of myofibroblasts (CM^MF^) [40] and NHDF (CM^NHDF^) was used as chemoattractant. Although CM^MF^ showed a higher chemoattractive potential than CM^NHDF^, which was already described previously [57], the number of invading tumor cells was dose-dependently lowered compared to mock-treated cells with a significant effect at the highest dose (Fig 6). In conclusion, these data along with the cytotoxicity and proliferation data indicate that CD also affects the invasive capacity of A375 melanoma cells.

### CD mediated cytotoxicity is independent of the cellular uptake

**Fig 7:**
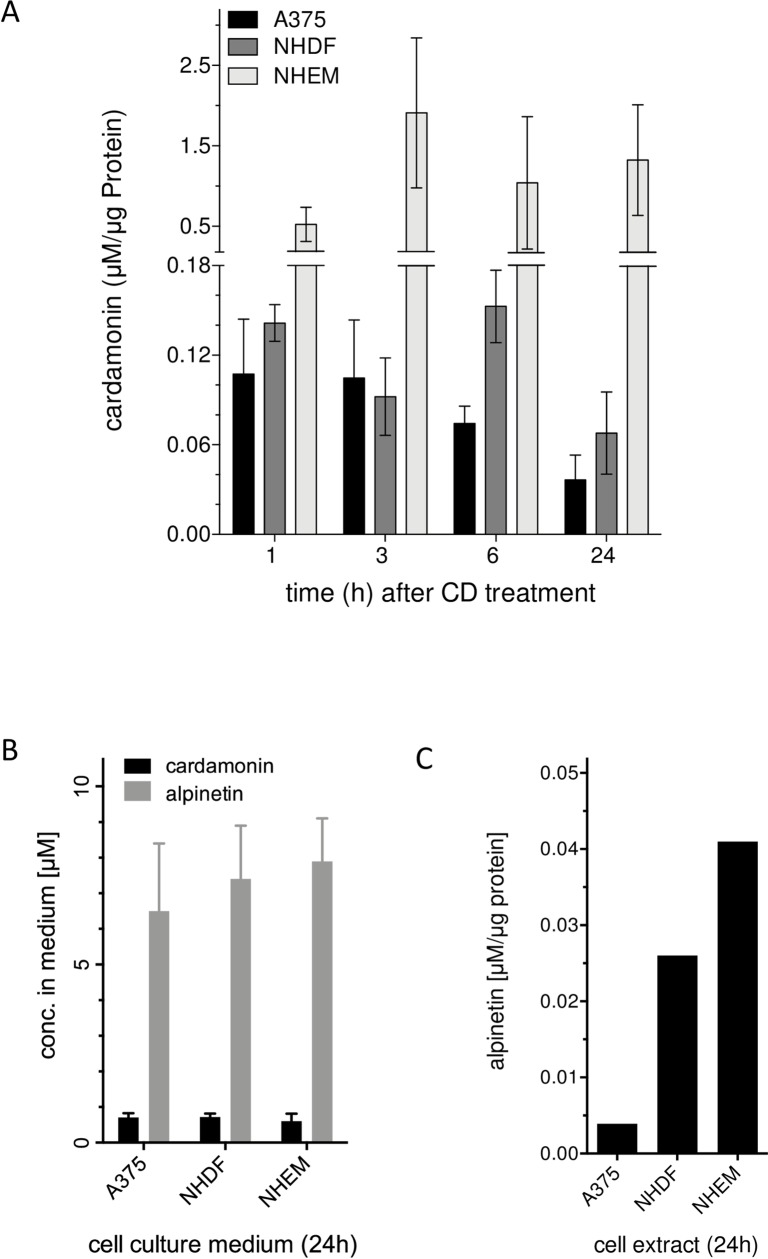
Selective CD toxicity on melanoma cells is independent of cellular uptake and oxidative stress. Melanoma cells, normal human dermal fibroblasts (NHDF), and normal epidermal melanocytes (NHEM) were incubated with 10 μM CD and the uptake of CD (A) and extracellular (B) and intracellular (C) alpinetin formation, respectively, were measured with an established HPLC method. The data (A, B) represent three independent experiments (n = 3) and (C) the data of 1 out of three independent experiments.

As the selective cytotoxicity of CD may depend on differences in the intracellular amount of the chalcone, the CD uptake was measured (Fig 7A). These studies were performed with an HPLC method clearly indicating that the intracellular CD concentration differed between the three cell lines whereby the CD concentration was even higher in NHDF and NHEM compared to the tumor cells. During the time period studied (1–24 h) no statistically significant change of the amount of CD was found within each cell type, even though the tumor cells showed an apparent moderate time dependent decrease of the CD amount until 24 h, with the highest concentration at 1 h post treatment (Fig 7A). These data suggest that melanoma cells are either metabolizing CD or extruding it faster than NHDF or NHEM. As a spontaneous cyclization of the chalcone CD to the flavanone alpinetin has been described [58,59], we checked the occurrence of alpinetin in the medium (Fig 7B) and in the cell extract after 24 h post incubation (Fig 7C). A high amount of alpinetin was detected in the cell culture medium (cell culture supernatant) of the three cell lines at 24 h after treatment the cells with CD indicating cyclization of CD to alpinetin (Fig 7B). In addition, a low amount of alpinetin could be detected in the extract of the cells after 24 h in only 1 out of 3 independent experiments with the lowest amount in A375 melanoma cells and the highest amount in melanocytes (NHEM) (Fig 7C). We thus can exclude that the selective cytotoxic effect in tumor cells is mediated by alpinetin. Interestingly, treatment with alpinetin directly showed that alpinetin was not taken up, neither by the tumor cells nor the normal (healthy) cells. In conclusion, it seems that at least a fraction of CD can (intracellularly) be metabolized to alpinetin.

### CD mediated cytotoxicity is independent of reactive oxygen species (ROS) and heme oxygenase-1 (HO-1) expression

**Fig 8:**
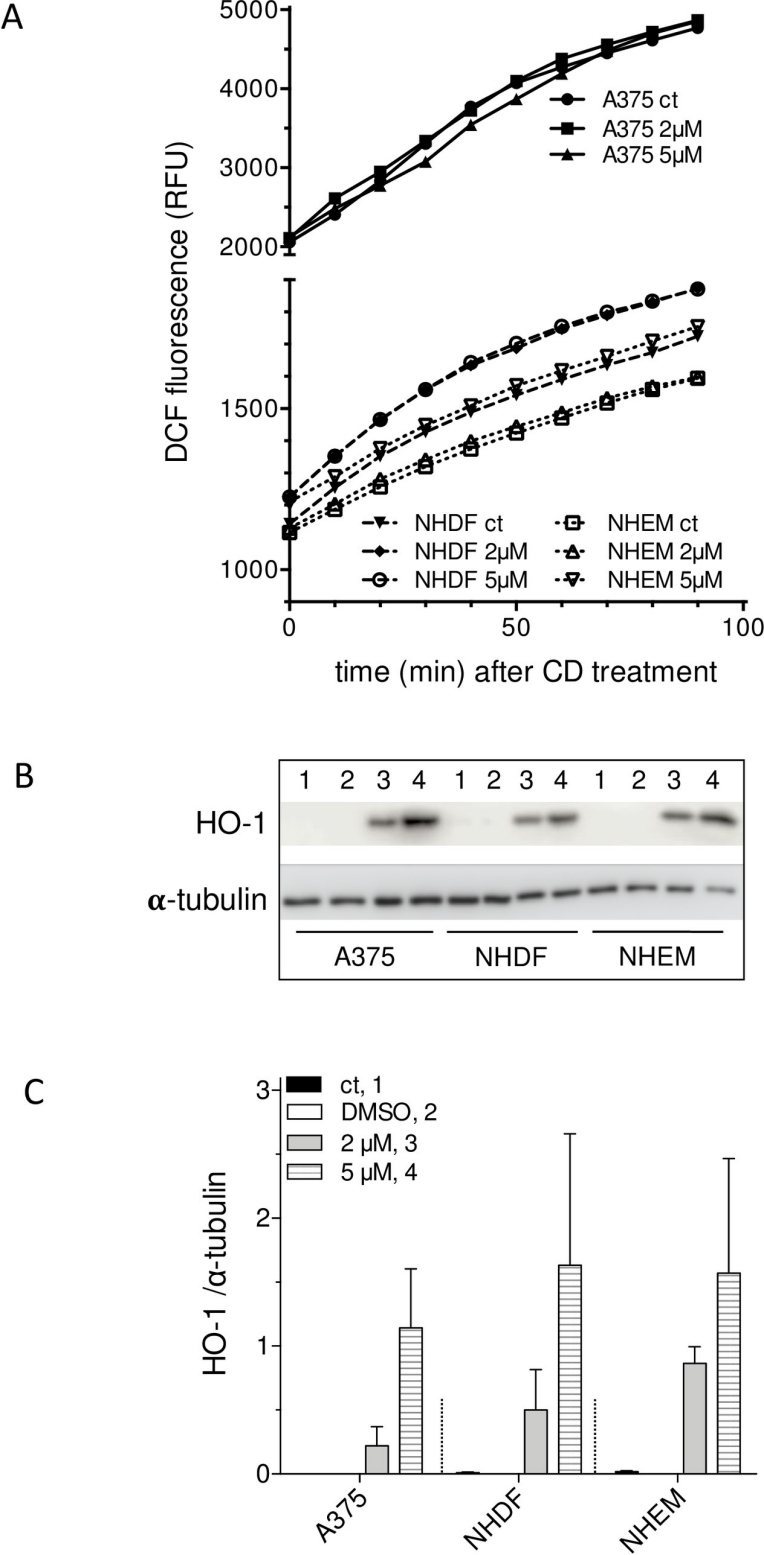
CD selectivity is independent of oxidative stress and HO-1 expression. For ROS detection (A), melanoma cells, normal human dermal fibroblasts (NHDF), and normal epidermal melanocytes (NHEM) were pretreated with H_2_DCF-DA followed by treatment with 2 and 5 μM CD. The DCF fluorescence as measure of the intracellular ROS level was determined every 10 min for 1.5 hours. The data (A) represent three independent experiments (n = 3), whereby SEM are not delineated because of better clarity. In addition, the three cell types were untreated (ct), mock-treated (DMSO), and treated with 2 or 5 μM CD for 24 h. After treatment, cells were lysed and prepared for Western blots to detect HO-1 protein levels. A representative Western blot (B) and the densitometric analysis (C, mean intensities ± SEM, n = 3) of the ratio of HO-1 to tubulin expression (loading control) are shown.

Others and our group pointed out that the cytotoxic effect of anticancer drugs on tumor cells is often mediated by their capability to further increase the intracellular ROS concentration which was already higher in tumor cells [60] resulting in oxidative damage. In a few publications it was shown that chalcones increase intracellular ROS level [39,61,62] which was measured amongst others with the fluorescent dye DCF. The peroxide sensitive DCF is widely used for detection of intracellular ROS, although its use has some drawbacks. For example, the fluorescence of DCF may also depend on the reaction with other intracellular compounds and not exclusively depend on ROS [63–65]. If adequate controls exist, the DCF assay nevertheless is a suitable tool to measure intracellular ROS amounts [66,67]. We asked whether the CD uptake significantly affects the intracellular ROS level subsequently being responsible for the cytotoxicity. The ROS level was measured every 10 min within 90 min directly (time point 0) after treatment the cells (Fig 8A). Consistent with earlier studies [57,68,69], the basic ROS level of the tumor cells is considerably higher than the level of the non-transformed cells. As the cells were pre-incubated with the ROS sensitive dye prior to treatment with CD at concentrations of 2 or 5 μM CD or mock-treatment, an increase in the DCF fluorescence was measured over the studied time period. That is based only on the order of the used substances. Yet, neither melanoma nor normal (healthy) cells showed a significant difference in the ROS amount between treated and mock-treated cells (ct) (Fig 8A). Thus, our data obtained with the fluorescent dye DCF negate the hypothesis that CD results in increased ROS formation.

Previous data demonstrated that the electrophilic potential of chalcones resulted in heme oxygenase-1 (HO-1, HMOX1) expression via Keap1/Nrf2 [42,70], which protects against inflammation, oxidative stress and other stress stimuli. Furthermore, HO-1 overexpression was described to impair tumor progression of non-small cell lung carcinoma cells [71], hepatocellular carcinoma cells [72], and triple-negative breast cancer cells [73] resulting in apoptotic cell death. In that context, HO-1 expression was investigated in the three cell lines (Fig 8B and 8C). A375, NHDF, and NHEM monolayer cell cultures were treated, untreated (ct) or mock-treated (DMSO) with two concentrations of CD. A dose-dependent expression of HO-1 was measured for all cell types at 24 h post treatment versus untreated or mock-treated cells (Fig 8B). The densitometric analysis indicated no significance between the HO-1 expression profiles of the 3 cell types (Fig 8C) supporting the conclusion, that the selective cytotoxicity of CD appears to be independent of HO-1 expression. However, it cannot be excluded that the electrophilicity of the chalcone is responsible for its selective effect as other proteins than HO-1 may be affected which resulted in apoptotic cell death [37,39]. Further studies have to be performed in that regard.

### Cytotoxic effect of CD on melanoma cells is mediated by apoptosis

**Fig 9:**
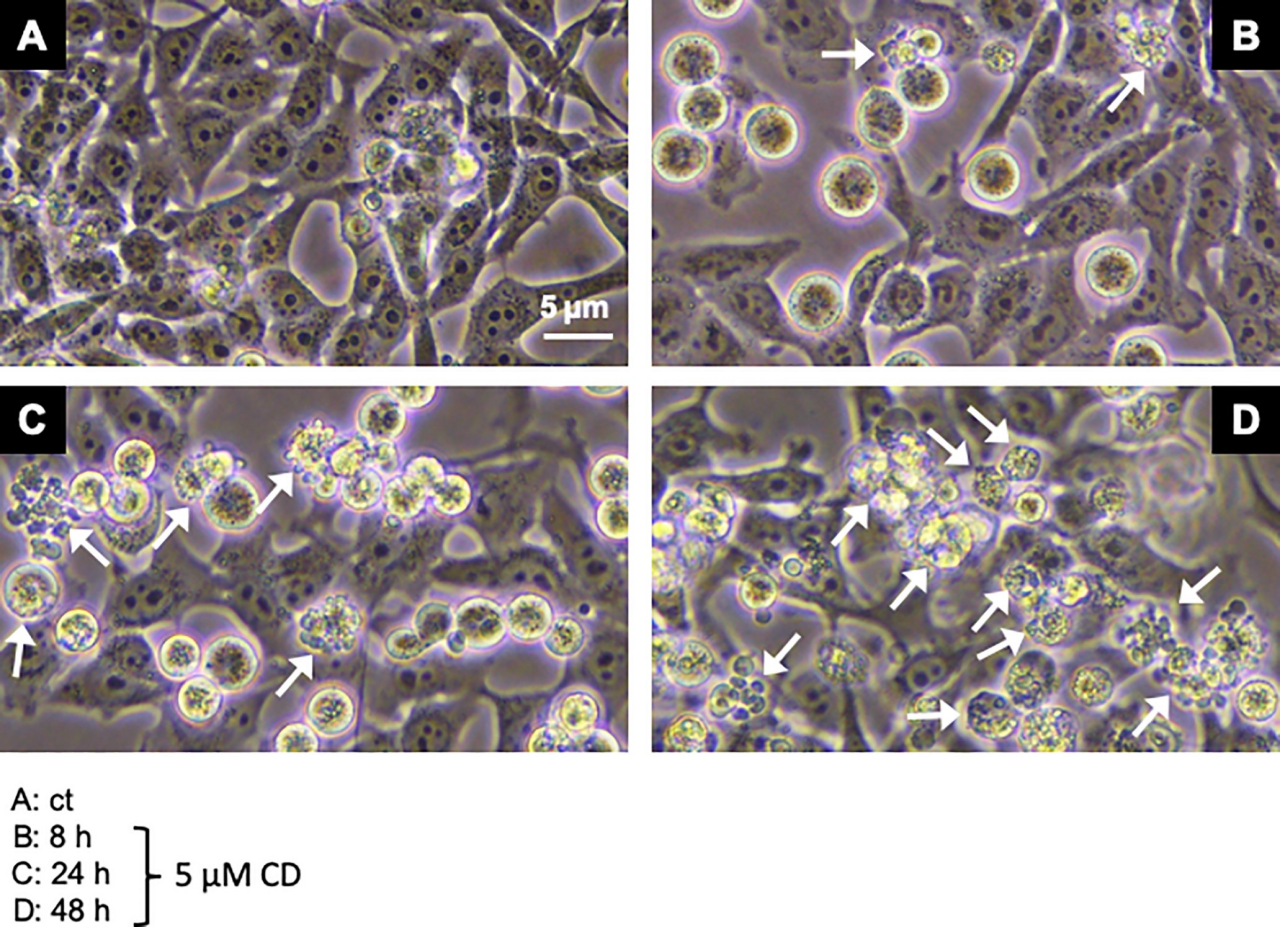
CD causes membrane blebbing in melanoma cells. The A375 cells were mock-treated (A, ct) or treated with 5 μM CD for 8 (B), 24 (C), and 48 (D) hours. Representative pictures of mock-treated and CD treated cells were taken by phase-contrast microscopy with a Nikon Eclipse Ts2 microscope using the Digital Sight DS-L3 camera. The white arrows indicate a time-dependent increase in membrane blebbing.

As it was already shown that CD and other chalcones such as xanthohumol or isoliquiritigenin stimulate apoptosis in some cancer types [30,38,74], the question was addressed of whether CD induces apoptosis in human melanoma cells as nothing was found so far in PubMed. Membrane blebbing, caspase-3 activation, and poly(ADP-ribose) polymerase 1 (PARP) cleavage, three key features of apoptosis [75], were studied as well as the effect of the pan caspase inhibitor z-VAD-FMK, a cell permeable fluoromethyl ketone (FMK)-derivatized peptide [76]. The treatment of A375 melanoma cells with a toxic concentration of 5 μM CD resulted in a time-dependent increase in membrane blebbing as first indication of apoptosis (Fig 9; white arrows) which was monitored by phase-contrast microscopy. Untreated (ct) melanoma cells exhibited the typical cellular morphology with large and round nuclei (Fig 9A). At 8 h post treatment with CD, a larger number of cells became round and detached (Fig 9B) followed by an increasing number of cells with membrane blebbing at 24 and 48 h (Fig 9C and 9D).

**Fig 10:**
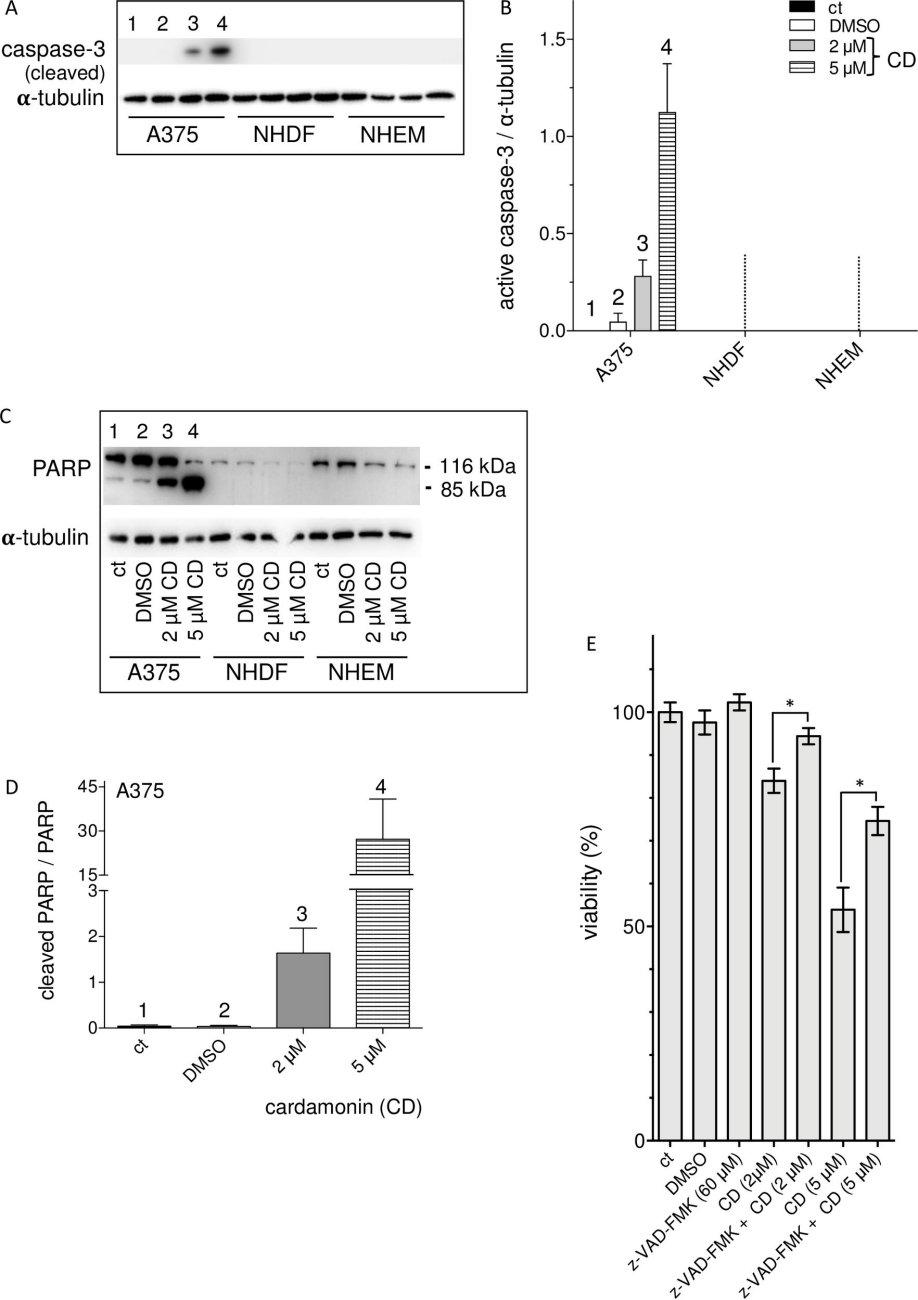
CD selectively induces apoptosis in melanoma cells. A375 melanoma cells, normal human dermal fibroblasts (NHDF), and normal epidermal melanocytes (NHEM) were untreated (ct), mock-treated (DMSO), and treated with 2 or 5 μM CD for 24 h. Cells were lysed and subjected to Western blot analysis to detect active (cleaved) caspase-3 (A, B) and uncleaved (active, 116 kDa) and cleaved PARP (85 kDa), respectively (C, D). A representative Western blot (A and C) of 1 out of three independent experiments and the densitometric analysis (mean intensities ± SEM, n = 3) of the ratio of caspase-3 to tubulin expression (loading control) (B) and of the ratio of cleaved PARP to (active) PARP in melanoma cells (D) are shown. Tubulin was used as loading control. The data (E) show the effect of the pan caspase inhibitor z-VAD-FMK on cell viability. The melanoma cells were untreated (ct), mock-treated (DMSO), z-VAD-FMK treated, CD treated or pretreated with the caspase inhibitor for 4 h at a concentration of 60 μM prior to treatment with 2 and 5 μM CD for additional 24 h. Thereafter the viability of the cells was measured with the MTT assay. The column graph is representative for 1 out of three independent experiments (n = 3) with mean and SEM indicating the values of three independent samples for each treatment. The level of significance was calculated (Student’s t-test) with *p<0.05.

As membrane blebbing is described to be dependent on a caspase-8 (extrinsic pathway) or caspase-9 (intrinsic pathway) mediated activation of caspase-3 [75,77], the amount of the active caspase-3 (cleaved caspase-3) was measured by Western blot (Fig 10A). Compared to untreated (ct) or mock-treated (DMSO) tumor cells, a concentration of 2 or 5 μM CD resulted in a strong caspase-3 signal at 24 h after treatment, whereby the intensity of the signal is dose-dependent as indicated by densitometric analysis (Fig 10B). Interestingly, healthy fibroblasts (NHDF) and melanocytes (NHEM) did not show caspase-3 activation at both concentrations.

A down-stream target of the active caspase-3 is the DNA repair-initiating enzyme PARP, which will be cut in two inactive fragments being a marker for apoptosis. PARP and poly(ADP-ribosyl)ation have been shown to be important for the regulation of many cellular processes such as DNA repair, chromatin functions and genomic stability [78]. Again, the three cell lines were untreated (ct), mock-treated (DMSO), and CD treated and active (116 kDa) and inactive (cleaved, 85 kDa) PARP detected by Western blot (Fig 10C). A dose-dependent increase in the amount of cleaved PARP (85 kDa band) was observed only in melanoma cells reflected by the densitometric analysis (Fig 10D), whereas no PARP cleavage was detected in NHDF and NHEM. Interestingly, the A375 melanoma cells have a significant higher base level of active PARP (Fig 10C) than the normal (healthy) cells. The much higher amount of PARP in the tumor cells versus normal cells is a phenomenon observed in numerous other tumor cells or tumor tissues, for example breast tumors [79], gastric cancer [80], acute myeloid leukemia [81], primary cervical tumors and cervical cancer cell lines [82] which was described to be associated with tumor progression and metastasis as well as a poor prognosis [83], whereby the mechanisms leading to that high PARP level have not been completely understood until now. Although the data obtained so far indicate that CD has a selective cytotoxic effect on A375 melanoma cells which may be mediated by apoptotic processes, a direct link between cytotoxicity and apoptosis could not be drawn so far. In that context, the pan caspase inhibitor z-VAD-FMK was used to prevent apoptotic processes [76] and to possibly counteract the CD mediated decrease in cell viability (Fig 10E, S2 Fig). The inhibitor z-VAD-FMKA had no toxic effect on the A375 melanoma cells at the used and earlier described concentration [76] compared with untreated or mock-treated cells. A dose-dependent decrease in viability of the melanoma cells was observed after treatment the cells for 24 h with CD alone compared with untreated, mock-treated or z-VAD-FMK treated cells. However, a combination of the inhibitor z-VAD-FMK and CD resulted in a significant higher viability of the tumor cells compared with only CD treated cells (Fig 10E). In conclusion, the selective cytotoxic activity of CD on the A375 melanoma cells was clearly shown to be mediated by induction of pro-apoptotic processes.

## Discussion

Even though the treatment modalities and the prognosis of early stage melanoma have improved in recent years and many more therapeutical approaches are currently under investigation, about half of the patients do not have long-term benefits from the current therapies and especially the overall prognosis for metastatic melanoma is still poor [3,8,9]. Although it currently seems that the treatment of metastatic melanoma could be revolutionized by personalized targeted therapy approaches [84], such an approach also bears a significant risk regarding the development of drug resistance or overstimulating the immune system [18,85,86]. On the basis of enormous therapy costs [87], the use of dietary constituents for chemoprevention is still in focus based on the knowledge that about 30% of all cancers are related to nutrition and foods [87,88]. There are some reported evidences showing association between specific foods and cancer [88,89]. Important questions arising in that context relate to whether food components really lower cancer incidence and interfere with tumor promotion or tumor progression. One class of compounds answering that questions are the chalcones representing a group of the polyphenolic family and being described to have a broad spectrum of biological and biochemical activities [21]. In recent years, numerous publications with synthetic chalcones have described anticancer effects on murine and human melanoma cells [23,25] and others [24,26]. Some of the synthetic hydroxychalcones exerted a cytotoxic effect on murine melanoma cells in the range of 25 to 100 μM studied over 72 h [23]. In addition, some of the synthesized fluorochalcones significantly lowered cell viability of human A375 melanoma cells with IC_50_ values ranging from 5 to 10 μM measured at 48 h after treatment [25]. However, a potential cytotoxic effect of the synthetic chalcones on normal (healthy) cells was not tested [23–26], but cannot be excluded. In our study, we tested the effect of the natural chalcone CD on the viability of A375 melanoma cells and compared the data to those of melanocytes (NHEM) and human dermal fibroblasts (NHDF). Compared to the synthetic chalcones, CD seems to be more effective on the A375 tumor cells as the IC_50_ value for 48 h treated cells was calculated by non-linear curve fit analysis to be 3.89 μM (based on data of Fig 2) and for 96 h treated cells to be 2.43 μM CD (Fig 3C). In addition, the effect of the natural chalcones xanthohumol (XN) and isoxanthohumol (IXN) was evaluated on two melanoma cell lines with dissimilar molecular background, the murine B16-F10 and the human A375 cells. The treatment of both cell lines with both XN and IXN resulted in a dose-dependent decrease of cell viability with IC_50_ values (48 h treatment) of 8.70 (B16-F10) and 15.00 μM XN (A375) and similar IXN concentration for both cell lines ranging from 22.15 to 22.90 μM IXN [90]. Again, the effect of such XN or IXN concentrations on cell viability of normal (healthy) cells such as melanocytes was not tested. In our study, CD concentrations required for killing the tumor cells had no harmful effect on melanocytes and dermal fibroblasts. Depending on its concentration, the alpha,beta-unsaturated aldehyde 4-hydroxynonenal (HNE) was described to have both tumor growth stimulating and proliferation inhibiting properties [49,91]. We used HNE as model substance to compare it with CD. Surprisingly, melanocytes and dermal fibroblasts were more affected than the melanoma cells at concentrations ≥20 μM HNE. The effective doses are in line with previously published data indicating that doses around 50 μM HNE inhibit proliferation of HeLa tumor cells [92]. Moreover, it was shown that HNE loaded ß-cyclodextrin nanocarrier potentiate its antitumor effect in A375 melanoma monolayer cultures and in human reconstructed skin carrying melanoma cells [93]. CD was also shown to inhibit proliferation of non-small-cell lung cancer cells (NSCLC) [34] and SKOV3 ovarian carcinoma cells [35] with the greatest impact at 72 h at concentrations greater than 20 μM CD. However, those concentrations could not be used in our proliferation studies as they killed melanocytes and dermal fibroblasts as well. The proliferation of the melanoma cells studied herein was significantly lowered at 24 and 48 h with a concentration of 5 μM CD which did not affect the proliferation rate of healthy cells. Tumor invasion and metastasis represent a major challenge in context with an effective cancer therapy. In our study, concentrations of 2 and 5 μM CD lowered the number of invading melanoma cells. These data are in line with previously published data on NSCLC [34], on prostate cancer cells [37], and on triple negative breast cancer cells [36] demonstrating an anti-invasive activity of CD as well.

In order to find the cause for the CD selectivity in our study, the uptake/bioavailability of CD was studied. It was shown that the amount of CD in NHDF and NHEM was roughly equal to the amount or even higher than the CD amount in the melanoma cells over the studied time period, indicating that the CD selectivity cannot be explained by differences in uptake. The bioavailability of CD and xanthohumol was previously shown for human hepatocellular carcinoma cells, colorectal adenocarcinoma cells, and primary cultured hepatocytes [94] as well as in human and animal studies [27,32]. As a CD mediated increase in the amount of reactive oxygen species (ROS) and in the amount of Nrf2 activated heme oxygenase-1 (HO-1) was discussed in several publications [39,95], the ROS level and HO-1 expression was checked in melanoma cells and non-transformed cells. Even though the base level of ROS was higher in the melanoma cells versus NHDF and NHEM, CD did not affect the ROS level significantly compared to the mock-treated cell types. HO-1 overexpression in several cancer types was shown to reduce tumor size, to increase sensitivity of the cells for apoptosis inducing drugs, to reduce tumor burden in animal models, and to result in longer survival time of patients [72,73]. Therefore, it is discussed to use HO-1 as target for an anticancer therapy [96,97]. On the other hand, an overexpression of HO-1 in murine melanoma resulted in increase in proliferation, resistance to oxidative stress, and shortened survival of mice. Interestingly, a dose-dependent induction of HO-1 expression with similar amounts was detected for all three cell types in this study herein. That can be explained by the ROS-independent electrophilic reactivity of CD with Keap1/Nrf2, subsequently resulting in HO-1 expression [42,70,95], but does not explain the selective cytotoxicity of CD on the A375 tumor cells as the normal (healthy) cells did not show a CD initiated and apoptosis mediated cytotoxicity. However, it cannot be completely excluded that HO-1 may play a role in the CD dependent cytotoxicity as tumor cells and the normal (healthy) cells may react in another manner on HO-1 induction. Further studies focusing on proteomic and metabolomic approaches should also achieve greater clarity regarding the molecular targets and mechanisms underlying the CD selectivity. An important question to be answered was whether apoptotic cell death is responsible for the CD cytotoxicity of the melanoma cells. The observed time-dependent increase in membrane blebbing after CD treatment provided an initial indication that apoptosis may be involved which was substantiated by the dose-dependent increase in caspase-3 activity and PARP cleavage in the A375 melanoma cells, two major characteristics of apoptotic cell death. These data are in line with earlier published data showing a CD mediated increase in cleaved caspase-3 (active form) and PARP cleavage in glioblastoma cells [38], in prostate cancer cells [37], in triple negative breast cancer cells [36], and in colon adenocarcinoma cells [39]. Also xanthohumol was shown to induce apoptosis in human colon cancer cells [30].

In this study, we used the chalcone CD and compared its effect on the metastatic melanoma cell line A375 with its effect on normal (healthy) cells (S3 Fig). We could show for the first time that CD treatment resulted in a selective and apoptosis-mediated increase in cytotoxicity of tumor cells whereas the viability of melanocytes and fibroblasts was hardly affected.

## Supporting information

S1 Fig Significant decrease of the viability of A375 melanoma cells after CD treatment. The data are related to Fig 2. Subconfluent melanoma cells were incubated with different CD concentrations for 24, 48, 72, and 96 h and viability measured with the MTT assay. The viability is shown as a percentage of mock-treated control (ct), which was set at 100%. Three (n = 3 for 0, 24, 48, 96 h) and four (n = 4 for 72 h) independent experiments were performed.
(TIFF)

S2 Fig CD selectively induces apoptosis in melanoma cells. The data are related to Fig 10E. The A375 melanoma cells were untreated (ct), mock-treated (DMSO), z-VAD-FMK treated, CD treated or pretreated with the caspase inhibitor for 4 h at a concentration of 60 μM prior to treatment with 2 and 5 μM CD for additional 24 h. Thereafter the viability of the cells was measured with the MTT assay. The column graph is representative for 1 out of three independent experiments (n = 3) with mean and SEM indicating the values of three independent samples for each treatment. The level of significance was calculated (Student’s t-test) with *p<0.05.
(TIFF)

S3 Fig Selective effect of cardamonin on melanoma versus normal (healthy) cells. The chalcone cardamonin (CD), being a secondary plant constituent, has received growing attention due to its potential benefits to human health. In this study, it was shown that cardamonin exerts a selective cytotoxicity resulting in apoptosis of melanoma cells, whereas the viability of melanocytes and fibroblasts was hardly affected at such concentrations. This study highlights that cardamonin may be a valuable tool in anticancer therapies.
(TIFF)
