# Supplementary figures and images for: In vitro selective cytotoxicity of the dietary chalcone cardamonin (CD) on melanoma compared to healthy cells is mediated by apoptosis
Source: PLoS One. 2019 Sep 25;14(9):e0222267. doi: 10.1371/journal.pone.0222267 (PMC6760786; doi:10.1371/journal.pone.0222267)

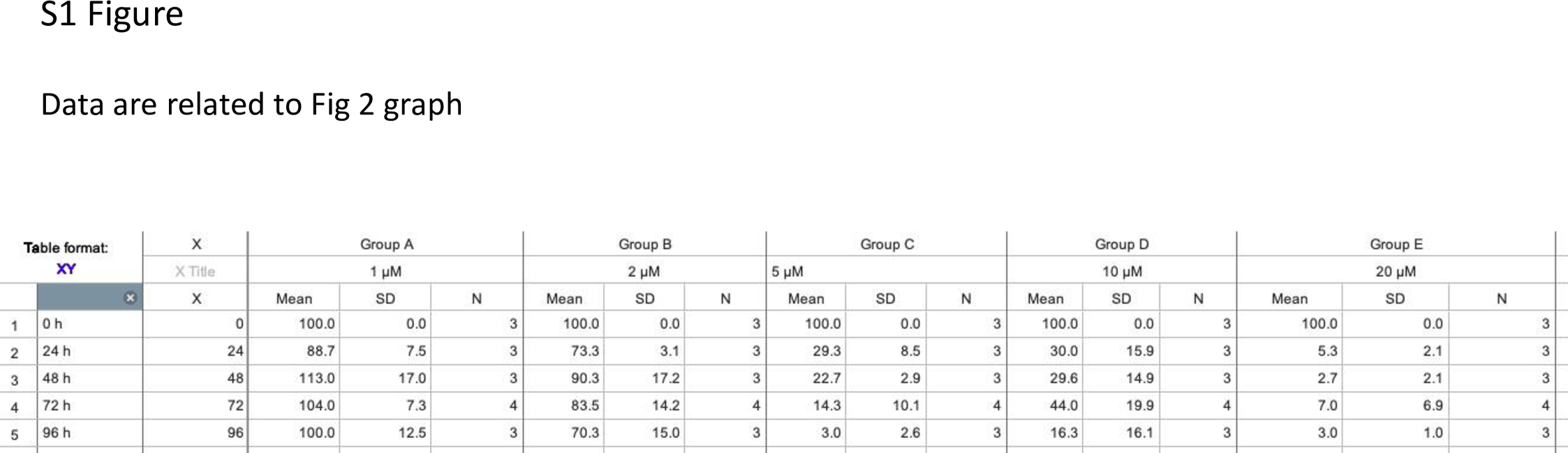

Supplement: S1 Fig — The data are related to Fig 2. Subconfluent melanoma cells were incubated with different CD concentrations for 24, 48, 72, and 96 h and viability measured with the MTT assay. The viability is shown as a percentage of mock-treated control (ct), which was set at 100%. Three (n = 3 for 0, 24, 48, 96 h) and four (n = 4 for 72 h) independent experiments were performed. (TIFF) [file pone.0222267.s001.tiff]

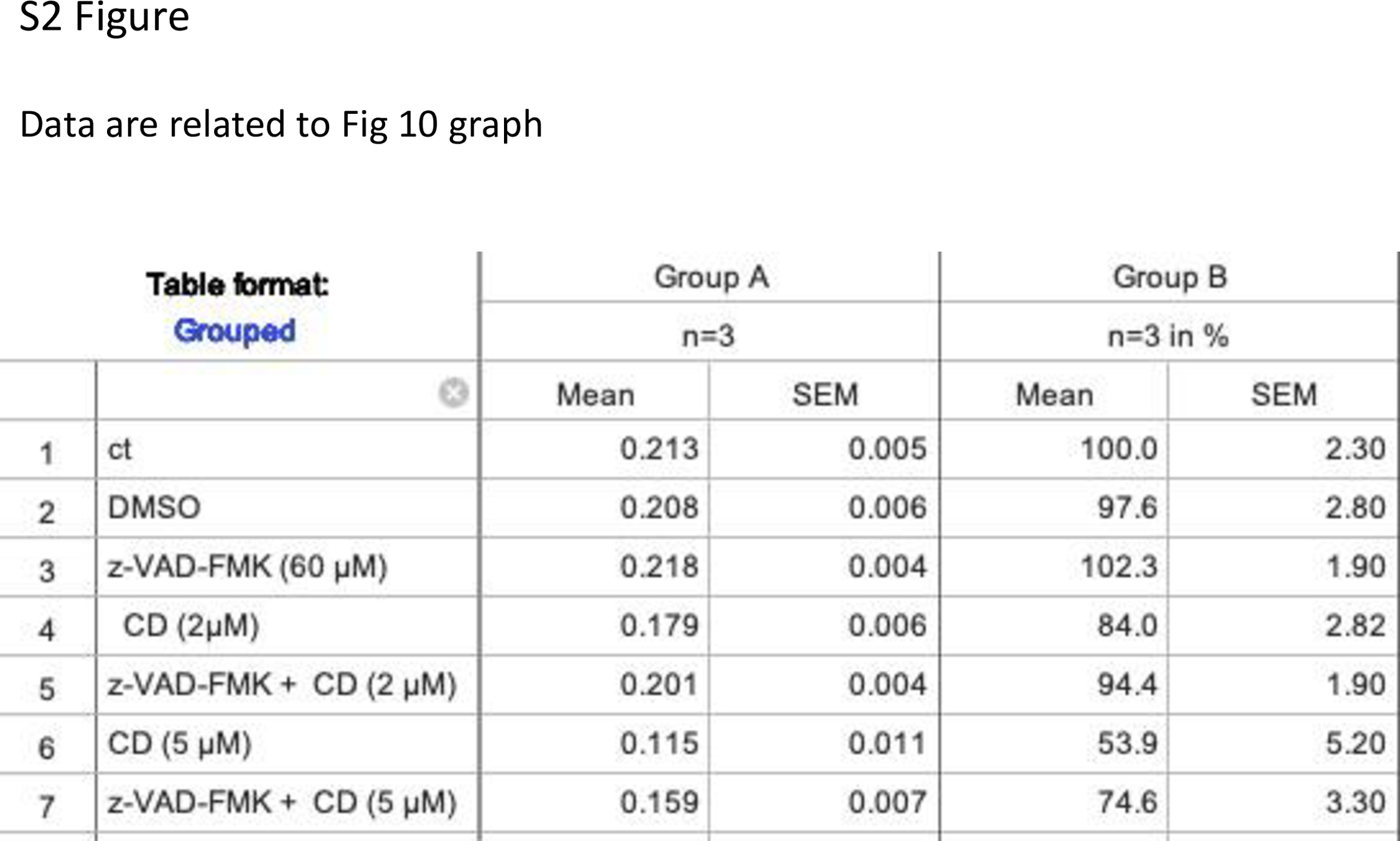

Supplement: S2 Fig — The data are related to Fig 10E. The A375 melanoma cells were untreated (ct), mock-treated (DMSO), z-VAD-FMK treated, CD treated or pretreated with the caspase inhibitor for 4 h at a concentration of 60 μM prior to treatment with 2 and 5 μM CD for additional 24 h. Thereafter the viability of the cells was measured with the MTT assay. The column graph is representative for 1 out of three independent experiments (n = 3) with mean and SEM indicating the values of three independent samples for each treatment. The level of significance was calculated (Student’s t-test) with *p<0.05. (TIFF) [file pone.0222267.s002.tiff]

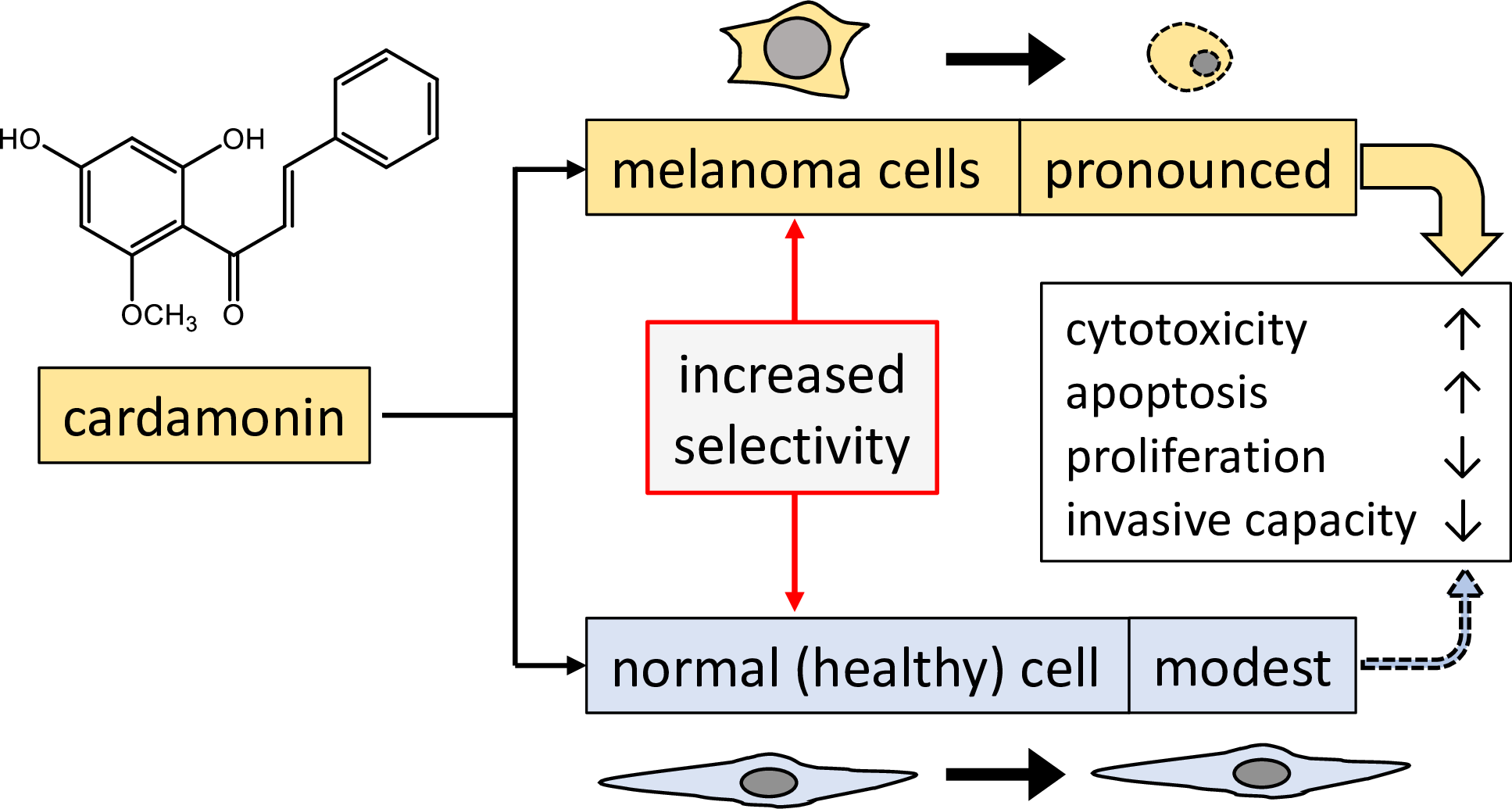

Supplement: S3 Fig — The chalcone cardamonin (CD), being a secondary plant constituent, has received growing attention due to its potential benefits to human health. In this study, it was shown that cardamonin exerts a selective cytotoxicity resulting in apoptosis of melanoma cells, whereas the viability of melanocytes and fibroblasts was hardly affected at such concentrations. This study highlights that cardamonin may be a valuable tool in anticancer therapies. (TIFF) [file pone.0222267.s003.tiff]
